# Bytes the Dust: Normative Notions in Decommissioning Digital Doppelgängers

**DOI:** 10.1080/15265161.2024.2441708

**Published:** 2025-01-29

**Authors:** Andrew J. Barnhart, Giuseppe Comerci, Matthias Braun

**Affiliations:** University of Bonn

## INTRODUCTION

In recent debates on digital twins, much attention has been paid to understanding the interaction between individuals and their digital representations (Braun, 2021). Iglesias et al. (2025) shed new light on this debate, extending the reflection on digital doppelgängers—digital twins that try to replicate the psychological dimension of an individual. They argue that such copies may serve as valuable means to achieve legacy and relational aims left unaddressed due to the person’s death. Against this background, we discuss how far we may better understand the implied normative aspects by considering them in terms of the represented person’s death. Specifically, we ask how we can and should, in normative terms, deal with a digital twin as a representation of a person after their death.

Here, we consider the decommissioning of such technology. We define decommissioning as the withdrawal, dismantling, or rendering the doppelgänger incapable of serving its original aims. We hypothesize that the way in which these digital doppelgängers ought to be decommissioned may depend upon whether they are viewed either as a proxy or as an extension of personhood. By proxy, we mean a stand-in for an individual by replicating their decisions and style without embodying their personal identity or subjective experience; something that makes decisions on your behalf but is not you (Braun and Krutzinna 2022). What is left behind is akin to an artifact owned by you. Whereas an extension of personhood can mean extending aspects of an individual’s identity and relational presence beyond death by reflecting their values, projects, and relationships; something that is/was a part of yourself. What is left behind is akin to an “informational corpse” (Öhman and Floridi 2018).

Answering this decommissioning question is necessary not only to respect the intended aims of those for whom the digital doppelgängers were created, but also to potentially respect certain social norms surrounding obsequies. Viewing digital doppelgängers either as proxies or extensions of personhood implies respective normative notions. For instance, the pursuit of any decommissioning strategy will require necessary and sufficient standards of informed consent, which may be difficult to parse given that not all individuals will view their digital doppelgänger in the same manner. The decommissioning of digital doppelgängers is thus enriched by moral nuances influenced by the perceptions we may have of this technology.

## DECOMMISSIONING STRATEGIES

Among the reasons that could lead to decommissioning are technical issues; for instance, bit rot may occur due to the physical deterioration of storage media or environmental factors affecting it. Excessive bit rot or physical failure could result in media decay, rendering digital doppelgängers unreliable (Corrado & Sandy 2017). Moreover, fulfilling the legacy purposes for which the digital doppelgänger was developed—such as posthumously completing the last part of an author’s novel—may also serve as rationale for decommissioning. Additionally, the surviving loved ones who may be interacting with the doppelgänger may no longer need it for grieving or other social relationship purposes, which then also raises questions about decommissioning. Similarly, should the surviving loved one also die, leaving behind the doppelgänger, then it would still require decommissioning.

Decommissioning digital doppelgängers can take two main forms: preservation or destruction. At first glance, there may be a preference for preserving a digital doppelgänger if it is viewed as an extension of personhood. Likewise, destruction could be seen as permissible if the doppelgänger is viewed as a proxy. However, such preferences and normative stances are not universally held, and it is not entirely clear which decommissioning strategy ought to be chosen.

Consider preservation: here, a first sub-strategy would be archiving. This entails creating and maintaining a repository for long-term storage and access to digital doppelgängers and data. Archiving (and preservation more generally) may be necessary given the rapid rate of technological change. The data, and doppelgängers themselves, may be inaccessible in just a few years time after they were created. Appropriately archiving provides a chance to maintain access to both the data and the doppelgänger. Archiving allows future generations to access prior iterations of digital doppelgängers. Should doppelgängers be viewed as proxies of a person’s decisions, style, or behavior, then archiving would serve more as a historical function. However, if the doppelgängers are viewed as an extension of a person, then archival functions as storage of these informational corpses, ostensibly creating and functioning as a kind of virtual cemetery. There, surviving loved ones could come and pay a visit online, similar perhaps to visiting a social media profile of the departed (Sisto 2020). This may imply that greater standards of archival are necessary for these virtual cemeteries.

A second sub-strategy of preservation would be repurposing. This entails repurposing digital doppelgängers for new applications, contexts, or aims which may be distinct from the original purposes. Examples may include research and development, education, or artistic purposes. Repurposing a digital doppelgänger still, in some sense, requires decommissioning and preservation of the original instantiation. Repurposing could be ethically justified if the digital doppelgänger is viewed as a proxy of the individual, where the information can be divested from the original purposes and used for more applications. One could use the data to help further develop better doppelgängers, for example. On the other hand, repurposing a doppelgänger under a personhood-extension view may be ethically more problematic, as it may be seen as a violation of the wishes or identity of the deceased individual. Consider the writer example from Iglesias et al. (2025), where the doppelgänger finishes the last bits of the author’s book. Repurposing that doppelgänger toward new literary works without the author’s explicit consent and authorization could raise serious ethical concerns as it may be perceived as an inappropriate use and violation of the author’s identity and legacy. For instance, suppose that the doppelgänger was altered in such a way that it could write explicit or adult content in the author’s style (assuming the author never wrote such material). While it is possible the doppelgänger could write explicit or adult content in the unique style and manner exactly like the original author, doing so could be a violation of the author’s wishes and identity under the person-extension view. More graphically, it might be akin to puppeteering an informational corpse for our own amusement.

The last strategy for decommissioning is destruction. Destruction of digital doppelgängers includes deleting data and software, as well as the disposal of the physical medium in which they are stored. Destruction is ideally a permanent strategy, one that, if done well, cannot be undone. It is also a state that preservation strategies try to actively avoid. Destruction could potentially be more acceptable if the digital doppelgänger is viewed as a proxy. In this way, the doppelgänger is more like an artifact that was owned by the individual, and its destruction may be justified, similar to destroying other possessions. Perhaps the more troubling conflict here centers in the person-extension view. Destroying an extension of personhood without proper memorialization may be seen as a form of digital desecration. In fact, as the digital doppelgänger may be seen as the very last remnant of an individual, some form of respect should be paid to it. For this reason, as physical bodies are honored with funerals, some kinds of ceremonies may be necessary when destroying these last remnants of the individual. However, destruction may be the preferred choice for many of those whom the digital doppelgängers represent, even if they do see it as an extension of themselves. The original person may wish that all related forms of their personhood are destroyed after serving their purposes.

## CONCLUSIONS

Exploring the relationship between representation and decommissioning strategies highlights different normative notions, which are summarized in Table 1. Archival strategies focus more on minimal standards of data storage, maintenance, and protection. Repurposing strategies directly engage with the means of divesting doppelgängers from their original purposes, some of which may be unavailable depending on the respective representational view. Lastly, destruction strategies hold normative notions of memorializing, focusing on acceptance of finality and proper handling of the deceased. This is not meant to be a full account of normative nations implied from viewing digital doppelgängers either as proxies or extensions of personhood. But these do stand out as further areas of needed exploration. Decommissioning, and how it is pursued, should not be forgotten in the ethical considerations of the technological life cycle of digital doppelgängers.

**Table 1.** A table showing normative notions involved when considering digital doppelgänger decommissioning strategies in combination with proxy or personhood-extension perspectives.

	Decommissioning strategies		
	Preservation		Destruction (memorializing)
	Archival (standards)	Repurposing (means)	
Proxy	Lower minimal standards	Greater available means for repurposing	Less need for memorialization
Personhood-extension	Higher minimal standards	Fewer available means for repurposing	Greater need for memorialization
